# Use of dulaglutide in a pregnant woman with type 2 diabetes until third trimester of pregnancy

**DOI:** 10.1007/s00592-024-02331-z

**Published:** 2024-08-10

**Authors:** Martina Molteni, Sara Lodigiani, Marsida Teliti, Mario Rotondi, Valeria Guazzoni

**Affiliations:** 1 Unit of Diabetology and Endocrinology, Medical-Oncologic Department, ASST Lodi, Lodi, Italy; 2 Department of Internal Medicine and Therapeutics, University of Pavia, Pavia, Italy; 3 Laboratory for Endocrine Disruptors, Unit of Endocrinology, Istituti Clinici Scientifici Maugeri IRCCS, Pavia, Italy

## Introduction

In the last decades, the number of pregnant women affected by pregestational type 2 diabetes (T2D) has increased. This is due to the rising global prevalence of T2D, to the decreasing age of the onset of T2D and to the higher mean age of the first pregnancy. In the US, the prevalence of diabetes mellitus in women of childbearing age was reported to be between 3.1 and 6.8%, with pregestational diabetes recorded in 1–2% of all pregnancies.

T2D in pregnancy carries on a high risk of both maternal and obstetrical complications including higher rates of miscarriage, preterm delivery and macrosomia. Some complications, such as preeclampsia, are even more frequent in pregnant women affected by T2D than in type 1 diabetes (T1D), due to the greater prevalence of coexisting cardiovascular risk factors in the first group of pregnancies.

Traditionally, insulin is the preferred pharmacological treatment in pregnant patients with both gestational and pregestational diabetes. However, recent evidences regarding the possible use of metformin in pregnant diabetic women have started to arise, although its use is still debated.

Glucagon-like-peptide-1 receptor agonists (GLP-1 RAs) are a class of medications currently not approved for the treatment of diabetes in pregnant women. Similarly, other new antidiabetic agents like dipeptidyl peptidase IV (DDP-4) inhibitors and gliflozins are not approved.

The concerns regarding the use of GLP-1 RAs in pregnancy are related to animal studies recording different adverse events in fetuses exposed to dulaglutide. In pregnant rats after exposure to high-dose dulaglutide reduced fetal weights associated with decreased maternal food intake were recorded and visceral malformation and skeletal defects were described. Muller DRP et al. recently reviewed the available data about GLP-1 RAs use in pregnancy: basing on animal studies and limited human data (only few accidentally exposed pregnancies) they recommendation against use of these medications in both pregnancy and lactation.

Interest in this case stems from the following clinical considerations:

(i) currently available literature data regarding the accidental use of GLP-1 RAs (liraglutide or dulaglutide) in pregnant women are limited to a few case reports [1–4] and one observational population-based cohort study [5];
(ii) at difference with previous reports, in which GLP-1 RAs was actually taken for a very limited gestational period (up to 4–12 weeks), in this case, dulaglutide was withdrawn only at the 34 gestational week;
(iii) recent recommendations strongly recommend avoiding GLP-1 RAs use in pregnant women and in women who are planning pregnancy. As a result, structured studies aimed at predicting possible clinical outcomes are currently not feasible. Thus, a description of every single case may be helpful in the clinical management of occasional situations like the here reported case.

To the best of our knowledge, few reports in literature reported an accidental use of GLP-1 RAs in the first trimester of pregnancy [1–5]. At difference, no data about the use of dulaglutide in the second and third trimester of pregnancy are available.

### Case description

We are here reporting the case history of a 42-year-old Caucasian pregnant woman affected by T2D, treated with dulaglutide in combination with metformin until the 34th gestational week because of an unplanned and unrecognized pregnancy.

The patient was diagnosed with T2D in 2013, when she was 32 years old. At the onset of disease antibodies of T1D were negative and C-peptide was preserved. No micro or macrovascular complications were detected in the follow up. She was also affected by obesity (pregestational body mass index 40 kg/m2) and combined dyslipidemia (therapy with atorvastatin started in 2018).

Originally, only a dietary approach was chosen, subsequently in two different intercurrent pregnancies a basal bolus insulin therapy was introduced, and finally from 2018 the patient was treated with metformin. Therapeutic compliance was quite good and the women attended a semestral follow-up program. In February 2022 glycated hemoglobin was not on target (7.2%, 55 mmol/mol) thus, dulaglutide 0.75 mg/week was added to metformin 2000 mg/day. At the scheduled follow-up visit of August 2022 metabolic control was getting worse (glycated hemoglobin was 7.5%, 59 mmol/mol) and body weight was increasing, so dulaglutide was upgraded to 1.5 mg/week. At this time, the patient was pregnant at 10th week of gestation but she was not aware of the pregnancy.

**Fig. 1:**
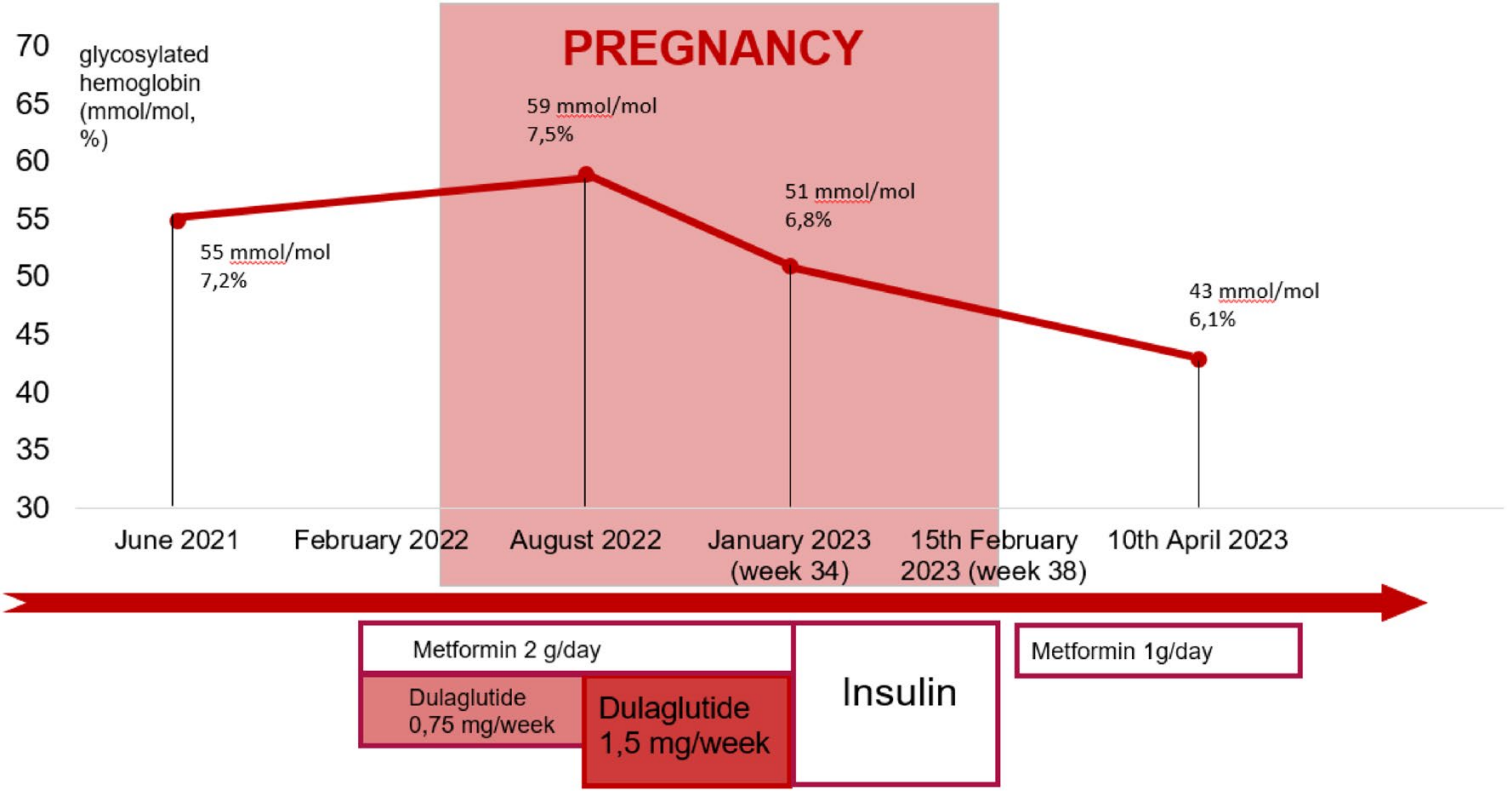
Timeline of therapeutic changes before, during, and after pregnancy and trend of glycemic control

In January 2023, she was referred to our Unit before the scheduled visit from the gynecological clinic because pregnancy was assessed. The patient did not consult a doctor until the 33rd week of gestation. This was her sixth pregnancy (G6P5). The fourth and the fifth pregnancies occurred after T2D diagnosis, basal bolus therapy was performed and sub-optimal glycemic control was achieved, the fifth pregnancy was complicated by fetal macrosomia, perinatal complications did not occur in any of the previous pregnancies. When the current pregnancy was discovered, her glycosylated hemoglobin was 6.8% (51 mmol/mol), not on target for pregnancy despite the ongoing hypoglycemic therapy. At this time, the patient was at her 34th week of gestation. Dulaglutide, metformin and atorvastatin were immediately stopped and she was switched to insulin therapy. For a few days we scheduled only basal insulin because postprandial values were well controlled, then an intensive basal bolus insulin therapy with dose titration was required to allow an appropriate glycemic control. The final insulin regimen was 3 units of Insulin Lispro 100 U/ml before breakfast, 4 units before lunch and dinner and 14 units of Insulin Glargine 300 U/ml. Only a small dose of fast-acting insulin was required because her adherence to the diet significantly improved when pregnancy was discovered. (Fig. 1).

The obstetrical ultrasound examination performed when pregnancy was discovered (33rd week of gestation) showed normal fetal anatomy, fetal growth was at 80°centile with abdominal circumference at 95°centile. Fetal biometry remained near the upper limit of normal in the following weeks. Fetal echocardiography was normal. Pregnancy was complicated by polyhydramnios and a borderline blood pressure was detected, so α-methyldopa was introduced. Microalbuminuria was normal, diabetic retinopathy was excluded and the patient did not show macrovascular complication of T2D. The woman was also treated with intravenous iron for anemia. Body weight gain during pregnancy was about 5 kg. The patient underwent an elective cesarean section at 38th weeks for breech presentation of the fetus. The newborn was a female, her weight was 3750 g. No congenital malformations were observed and no neonatal complications were detected, particularly no neonatal hypoglycemia was recorded.

The woman did not develop any complications during cesarean delivery and she did not breastfeed for choice, insulin was stopped immediately after the delivery and then, after a week post-partum, metformin therapy was restored due to raising fasting blood glucose. Limited to the short available follow up, the newborn is having a regular growth.

## Discussion

The present case description indicates that, even if dulaglutide had been used up to the third trimester of pregnancy, no fetal malformations nor neonatal complication occurred and no impact on fetal growth was observed. It is clear that a single case description does not allow drawing firm conclusions as to the potential risks related to the use of dulaglutide in pregnant women, also since data obtained from animal studies warned about the potential risk of dulaglutide-associated malformations. Unlike data from animal studies, in our case, dulaglutide did not affect organogenesis and skeletal development. However, it should be emphasized that malformations were recorded with very high dulaglutide plasmatic concentrations. Moreover, in previous studies, dulaglutide seemed to be related to fetal growth restriction due to reduced maternal food intake. This phenomenon did not occur in our patient, even though the prolonged use of dulaglutide throughout gestation could potentially be a risk factor for hyponutrition.

The pregnancy we described was otherwise complicated by polyhydramnios and fetal biometry was borderline for the diagnosis of large for gestational age. We do not have enough data to understand the possible contribution of dulaglutide exposure to the development of these complications, but these two conditions are likely to be related to obesity and poor glycemic control per se rather than to dulaglutide. Indeed, the patient displayed several risk factors for maternal/fetal complications other than exposure to GLP-1 RAs: first of all woman’s age, then obesity, suboptimal glycemic control, borderline elevated blood pressure, and contemporary exposure to statin (drug with well-known teratogenic effect). Among others, especially obesity of third degree could contribute to increase the risk of hypertension, macrosomia, polyhydramnios and cesarian section.

To the best of our knowledge, only four case reports described the effect of GLP-1 RAs accidentally used in the first weeks of pregnancy so far (i.e. before pregnancy was diagnosed) [1–4]. In these reports, fetal exposure to dulaglutide, exenatide or liraglutide in the first trimester did not affect pregnancy development and fetal outcomes. Moreover, during clinical studies on dulaglutide, few women experienced unplanned pregnancies during therapy with dulaglutide, GLP-1 RA was stopped and no neonatal complications were recorded [2. The here reported case is the first description of a prolonged use of dulaglutide up to the 34th week of gestation.

Recently, Minis et al. aimed to revise the literature for evidence-based guidance regarding the preconception use of GLP-1 RA. The Authors did not find adverse pregnancy or neonatal outcomes. However, they advise a precautional washout period of 4 weeks before attempting conception.

To further investigate the issue, Cesta et al. conducted a cohort study using nationwide population registries and databases from Finland, Sweden, Norway, Iceland, the United States, and Israel. In pregnant patients with pregestational T2D, periconceptional exposure (from 90 days before the first day of the last menstrual period to the end of the first trimester) to GLP-1 RAs and other second-line antidiabetics was not associated with an increased risk of major congenital malformations compared with insulin. Of note, despite including data from six countries, the sample size of infants exposed to specific second-line noninsulin antidiabetic medication classes remained relatively small during the study period [5].

However, it should be remembered that some recent animal studies evaluated the potential therapeutic use of these drugs also in terms of pregnancy complications. Recently, it was demonstrated that treatment with liraglutide in pregnant mice can attenuate hypertension offering a potential therapeutic option for preeclampsia even if its use was associated with decreased fetal weight. Moreover, recent studies detect that GLP-1 may have a role in the mechanism of compensation of pregnancy-related increase in glycemia and insulin resistance and that pregnancies complicated by gestational diabetes have an impaired response of postprandial GLP-1. This new knowledge opens the door to a possible physio-pathological beneficial effect of GLP-1 RAs, regardless of all the proper considerations about the safety of their use in pregnancy.

Despite the lack of major consequences due to dulaglutide use observed in our pregnant patient, no firm conclusion as to the safety of dulaglutide use in pregnant patients can be drawn. However, we think that description of the present case can be useful in clinical practice. Indeed, our experience might provide clinicians facing similar continuation (unintentional prolonged use of dulaglutide due to unawareness of pregnancy) some additional information.

In the here described case, we also discontinue metformin when pregnancy was discovered. We made this decision because at that time, even if Italian Medicines Agency had already approved metformin use in pregnant women, Italian position paper about this topic had not been published yet, current guidelines did not include metformin as a possible therapeutic option and scientific societies still recommended caution.

Finally, clinical cases like the one described here as well as others reported in literature, underline that clinical education of women affected by T2D with regards to pregnancy should be improved. Indeed, unplanned pregnancies in T2D diabetes are more frequent than in women with T1D, who are more often aware of the risks of poor pregnancy preparation. Moreover, women with T2D, are likely to be more often treated with new drugs (potentially affecting organogenesis) as compared to those with T1D. Thus, for women of childbearing age an effective periodical counselling session aiming to plan pregnancy only when optimal glycemic control is reached with safe therapies appears worthwhile, moreover drop out of diabetic young women should be avoided through close follow up program.

In conclusion, this case report represents the first description of an unintentional prolonged use of dulaglutide during pregnancy which turned out not to affect fetal development. Despite the use of GL1-RA until the third trimester of pregnancy, the newborn was healthy, and no malformations or neonatal complications occurred.
